# Commutability of Possible External Quality Assessment Materials for Cardiac Troponin Measurement

**DOI:** 10.1371/journal.pone.0102046

**Published:** 2014-07-07

**Authors:** Shunli Zhang, Jie Zeng, Chuanbao Zhang, Yilong Li, Haijian Zhao, Fei Cheng, Songlin Yu, Mo Wang, Wenxiang Chen

**Affiliations:** 1 Beijing Hospital and National Center for Clinical Laboratories, Ministry of Health, Beijing, China; 2 Chinese Academy of Medical Sciences and Peking Union Medical College, Beijing, China; 3 Department of Laboratory Medicine, Beijing Hospital, Ministry of Health, Beijing, China; 4 Department of Laboratory Medicine, Peking Union Medical College Hospital, Beijing, China; Emory University, United States of America

## Abstract

**Background:**

The measurement of cardiac troponin is crucial in the diagnosis of myocardial infarction. The performance of troponin measurement is most conveniently monitored by external quality assessment (EQA) programs. The commutability of EQA samples is often unknown and the effectiveness of EQA programs is limited.

**Methods:**

Commutability of possible EQA materials was evaluated. Commercial control materials used in an EQA program, human serum pools prepared from patient samples, purified analyte preparations, swine sera from model animals and a set of patient samples were measured for cTnI with 4 assays including Abbott Architect, Beckman Access, Ortho Vitros and Siemens Centaur. The measurement results were logarithm-transformed, and the transformed data for patient samples were pairwise analyzed with Deming regression and 95% prediction intervals were calculated for each pair of assays. The commutability of the materials was evaluated by comparing the logarithmic results of the materials with the limits of the intervals. Matrix-related biases were estimated for noncommutable materials. The impact of matrix-related bias on EQA was analyzed and a possible correction for the bias was proposed.

**Results:**

Human serum pools were commutable for all assays; purified analyte preparations were commutable for 2 of the 6 assay pairs; commercial control materials and swine sera were all noncommutable; swine sera showed no reactivity to Vitros assay. The matrix-related biases for noncommutable materials ranged from −83% to 944%. Matrix-related biases of the EQA materials caused major abnormal between-assay variations in the EQA program and correction of the biases normalized the variations.

**Conclusion:**

Commutability of materials has major impact on the effectiveness of EQA programs for cTnI measurement. Human serum pools prepared from patient samples are commutable and other materials are mostly noncommutable. EQA programs should include at least one human serum pool to allow proper interpretation of EQA results.

## Introduction

The measurement of cardiac troponin (cTn) has become an important clinical laboratory measurement because of its central role in the diagnosis of acute myocardial infarction (MI) [1]. Both cTnT and cTnI are specific and sensitive biomarkers of myocardial injury with necrosis. Currently, all cTnT assays are produced by a single manufacturer and assay results are comparable, whereas cTnI assays are produced by various manufacturers and the results are variable [2], [3]. This variability is undesirable for clinical use of the important biomarker and efforts are being made toward standardization of cTnI measurements [2], [4], [5].

Identification of variability of measurement results between assays and surveillance of the effectiveness of standardization is best accomplished through external quality assessment (EQA) (or proficiency testing) programs that use commutable samples [6]–[8]. EQA is now a common practice in laboratory medicine and nearly all clinical laboratories (assays) regularly participate in EQA programs; the samples used in EQA programs need to be commutable or the commutability needs to be known, otherwise the purpose of evaluating the comparability of different assays will not be fulfilled [9], [10]. However, for practical reasons, most current cTnI EQA programs use processed materials with unknown commutability and the interpretation of EQA results is often difficult. Our cardiac marker EQA programs using commercial control materials have shown between-assay discrepancies in cTnI results that are very different from that in the cut-off values (the 99th percentiles) of the assays (internal data and will be presented in this report), suggesting major noncommutability of the control materials. If this is the case, the magnitude of the noncommutability of the materials needs to be known and, when necessary and possible, more suitable materials need to be used in the EQA program.

Materials most likely to be commutable would be serum pools prepared according to the CLSI C37A guideline [11] which describes a rigorous protocol for the collection of blood from donors and the preparation of genuine serum pools for cholesterol. The C37A protocol has been validated or used in investigations to produce commutable samples for several other analytes [8], but it would hardly be applicable to the preparation of samples for cTnI measurement because detectable cTnI is primarily seen in MI patients. The best available approach to obtain materials likely to be commutable would be the use of leftover patient samples [8]. The commutability of such samples for cTnI measurement has been suggested [5], [12]. Because of the sample volume required in an EQA program, the preparation of the patient sample pools would involve a series of steps such as collecting, freezing storage, thawing, pooling, filtering, aliquoting, and re-freezing. Commutability of so-prepared materials for cTnI measurement has not been studied.

The use of patient samples, though possible, still poses significant difficulties especially in collecting samples in sufficient volume and with appropriate analyte concentrations. Combination of patient samples with more easily available materials that have acceptable commutability might be a practical approach for the EQA of cTnI. Other sources of possible EQA materials for cTnI may include purified analyte spiked in a serum matrix and animal sera. It has been reported that a cTn TIC complex purified from human heart tissue has reasonable commutability, though not totally commutable, among cTnI assays [5], [13]. A Standard Reference Material (SRM 2921) has been prepared from the TIC complex by NIST [14]. It has also been reported that cTnIs in big mammals share high homology with human cTnI and shows adequate responses to human cTnI assays [15], [16]. Among cTnIs in species, swine cTnI seems to have the most similar cross-reactivity to human cTnI antibodies [17]. The degree of commutability of these materials is currently unknown.

To interpret our EQA results and analyze the impact of noncommutability of samples on EQA programs, and in search for possible EQA materials for cTnI, in this study we evaluated the commutability of the control materials used in our EQA program, frozen serum pools prepared from patient samples, the NIST SRM 2921 diluted with human serum and swine sera from MI model animals.

## Materials and Methods

The study was a commutability study carried out according to a protocol as described in the CLSI C53A guideline [18]. The study involved measurement of prepared materials together with a set of individual patient samples with different cTnI assays. The mathematical relationships among the results of different assays for the prepared materials were compared with that for patient samples.

### Ethics Statement

The study involved use of leftover patient samples and animal serum samples. The leftover patient samples were all de-identified during the collection. It was also ensured that appropriate amount of serum was collected from each patient sample so that a certain volume was left for possible repetition of measurement. The use of patient samples in the present study has been reviewed and approved by the Ethics Committee of Beijing Hospital, Ministry of Health. The animal serum samples were stored swine sera from model MI animals that had been used in a previous study. The animals were induced with MI to test the effect of a traditional medicine (extracts of *salvia miltiorrhiza* and *carthamus tinctorius*) on post-MI coronary microcirculation. The study was conducted at the Research Center for Coronary Heart Disease, Fuwai Hospital and had been approved by the ethics committee of the institution.

### Individual Patient Samples

The individual patient samples used for the commutability study were leftover patient serum samples collected from the clinical laboratories of Beijing Hospital and Tongren Hospital. De-identified patient samples with measurable cTnI values (with a Beckmann Access assay at Beijing Hospital or a Siemens Centaur assay at Tongren Hospital) and sufficient leftover sample volume (sufficient for measurement in triplicate with 4 assays) were collected. A total of 75 samples were collected from 71 patients (36–92 years of age, 55 males) who were either admitted to the hospitals because of symptoms of suspected myocardial ischemia or hospitalized with diagnosed MI. Each of the samples was split into 4 aliquots and frozen at −86°C. All the samples were collected, aliquoted and frozen within 30 hours after blood drawn.

### Prepared Materials

Materials evaluated for commutability in the study included control materials used in our 2013 EQA program, frozen serum pools prepared from leftover patient samples, SRM preparations made by diluting the SRM 2921 with human serum and swine sera from MI model animals.

#### EQA materials

The EQA materials used in our 2013 cardiac marker EQA program were Bio-Rad Liquichek Cardiac Markers Plus Control LT control materials. Five levels of the materials (EQA L1-5) selected from Lots 23541, 23542, 23543, 29791 and 29792 were used for the program. Two levels (EQA L2 and L4) were evaluated as representatives for commutability.

#### Human serum pools

The human serum pools were also prepared from leftover patient serum samples collected from the two Hospitals. Possible volumes of leftover samples with cTnI values higher than 1 ng/ml (Beckmann Access assay or Siemens Centaur assay) were collected into tubes and frozen at −86°C every day. During a period of about 2 months, a total volume of approximately 200 ml of serum comprising 120 patient samples were obtained. The frozen aliquots of serum were thawed, pooled and tested for cTnI with Siemens Centaur CP assay. These primary pools were then diluted with a normal human serum pool, which was previously prepared and frozen-stored, to produce 5 patient serum pools (HSP L1-5) with cTnI values (Siemens Centaur CP assay) of approximately 8, 4, 2, 0.2 and 0.04 ng/ml, respectively. The pools were thoroughly mixed, filtered through 0.22 µm membranes, aliquoted in 0.8 ml into 2-ml cryogenic vials and stored at −86°C.

#### SRM preparations

Two levels of SRM preparations (SRM L1 and L2) were prepared by a serial dilution of SRM 2921 with a normal human serum pool. The cTnI concentrations of the preparations calculated from the assigned value [13] were 14.08 and 1.14 ng/ml, respectively. The preparations were aliquoted and frozen at −86°C.

#### Swine sera

Two cTnI positive swine sera were obtained as gifts from the Research Center for Coronary Heart Disease, Cardiovascular Institute & Fuwai Hospital, Chinese Academy of Medical Sciences. The sera were prepared from blood samples taken from model animals with MI induced by a balloon occlusion of the left anterior descending artery.

### Measurement of the Samples and Materials

The individual patient samples and prepared materials were measured with 4 cTnI assays including Abbott Architect (Architect), Beckman Access (Access), Ortho Vitros (Vitros) and Siemens Advia Centaur (Centaur). The cut-off values (99th percentiles) of the assays indicated in the assay instructions were 0.028, 0.04, 0.034 and 0.04 ng/ml, and the reportable ranges 0.01–50, 0.01–100, 0.012–80 and 0.006–50 ng/ml, respectively. The measurements were performed by the Abbott Shanghai Laboratory (Architect) and clinical laboratories of Beijing Hospital (Access and Centaur) and Beijing Haidian Hospital (Vitros). A detailed measurement protocol was prepared and understood by all the laboratories. The samples and materials were so labeled that the prepared materials were interspersed between patient samples. The whole set of samples was divided into 3 subsets for measurements in 3 days. Patient serum pool level 4 was included in each subset for the estimation of within-laboratory total CV. The samples were shipped on dry ice to laboratories outside the Hospital. On the day of measurement in each laboratory, a subset of samples were allowed to stand at room temperature for 30–60 minutes for thawing and mixed for 30 minutes on a hematology mixer. The samples were briefly centrifuged to collect the sample volumes which were fairly sufficient for the measurements. The samples were measured in triplicate and the order of measuring samples was reversed between the replicates. Calibrations were performed every day.

### Data Analysis

Individual patient samples with measured cTnI concentrations out of any of the reportable ranges of the 4 assays were excluded for data analysis. Twelve such samples were excluded, eight of which were too low to be detectable with the Vitros assay and four were too high with the Centaur assay. One sample had incomplete data for the Vitros assay and another showed an exceptionally high value with the Access assay and these two samples were also excluded. The remaining 61 samples were used for the analysis of between-assay correlations and the evaluation of the commutability of prepared materials. The between-assay slopes and intercepts for the 6 assay pairs formed by the 4 assays were estimated with Passing-Bablok regression on the basis of the mean values of the triplicate measurements. The Pearson correlation coefficients were also calculated. For commutability evaluation, the measurement results were logarithm-transformed because of the heteroscedasticity of the data. The transformed data were analyzed with Deming regression and 95% prediction intervals were calculated for each pair of assays, using formulas given in the CLSI C53A document [18]. The commutability of the prepared materials was evaluated by comparing the logarithms for the materials with the limits of the intervals. For the estimation of matrix-related biases for noncommutable materials, the predicted logarithms were back-transformed and relative differences of measured values from predicted values were calculated. The Passing-Bablok regressions were performed with Analyse-it and all other analyses with Microsoft Excel.

## Results

### Results from EQA program

Our institution (National Center for Clinical Laboratories) as a national EQA provider distributes 5 samples biannually to applicant laboratories for the EQA of cardiac markers. The samples used have been commercial control materials. For the first EQA event in 2013, 418 laboratories using 32 assays participated in the program. The major participant assays were Access (126 user laboratories), Centaur (63), Architect (60) and Vitros (13). **Table 1** shows the assay peer group means and inter-laboratory CVs of cTnI measurements with the assays. The peer group means varied approximately 2 to 10 fold. The inter-laboratory CVs were also variable.

**Table 1:** Assay group means and inter-laboratory CVs of cTnI measurements in the 2013 EQA program.

		Mean, ng/ml					CV, %				
Assay	N^a^	L1^b^	L2^b^	L3^b^	L4^b^	L5^b^	L1^b^	L2^b^	L3^b^	L4^b^	L5^b^
Access	126	0.04	0.69	3.16	0.50	2.17	25.0	17.4	16.8	18.0	18.4
Architect	60	0.16	6.56	32.59	5.02	22.74	18.8	7.9	8.3	8.0	6.8
Centaur	63	0.07	1.64	9.43	1.10	5.65	14.3	11.6	12.6	10.9	12.0
Vitros	13	0.08	3.35	18.58	2.65	12.39	12.5	3.6	6.2	4.2	4.5

a Number of laboratories.

b EQA material level 1 through 5.

### Measurement Results for Patient Samples

In the present study, we measured patient samples with the 4 assays for the purpose of evaluating the commutability the EQA materials and other materials. The medians and ranges of the measured cTnI concentrations and measurement precisions for the patient samples are shown in **Table 2**. The median cTnI concentrations of the 61 samples obtained with different assays varied from 1.475 ng/ml to 2.198 ng/ml. The ranges of cTnI concentrations were approximately 1000-fold wide (e.g. 0.026∼25.472 ng/ml for Access assay) and the standard deviations of the triplicate measurements were evidently proportional to the concentration levels. The median within-run CVs for the assays were between 1.0% and 5.9% and the within-laboratory total CVs as estimated from the results of human serum pool level 4 ranged from 1.3% to 5.1%.

**Table 2:** Patient serum sample cTnI concentrations and measurement CVs with different assays.

Assay	cTnI concentration, median (range), ng/ml	Within-run CV, median (range), %	Total CV^a^, %
Access	1.475 (0.026∼25.472)	5.9 (0.5–14.5)	4.9
Architect	1.735 (0.016–23.534)	2.5 (0.1–13.2)	2.2
Centaur	2.198 (0.033–41.285)	2.2 (0.0–15.1)	5.1
Vitros	1.522 (0.027–23.944)	1.0 (0.0–11.8)	1.3

a Estimated from measurement results of patient serum pool level 4.

**Figure 1:**
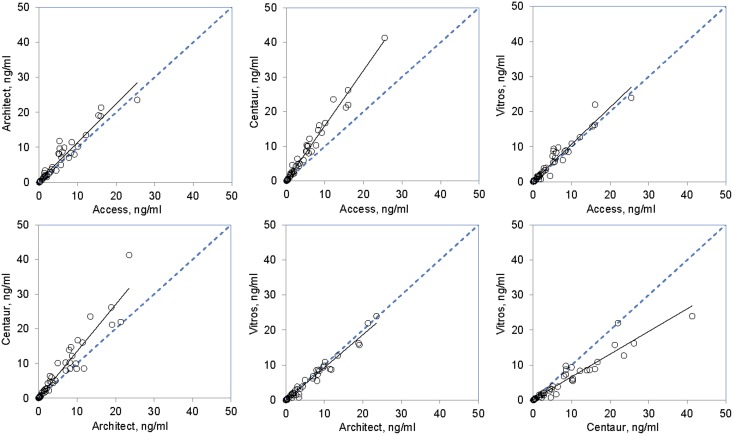
Scatter plots of cardiac troponin I (cTnI) concentrations measured with different assays. The cTnI concentrations of 61 patient samples were measured in triplicate with 4 assays including Abbott Architect (Architect), Beckman Access (Access), Ortho Vitros (Vitros) and Siemens Advia Centaur (Centaur). The means of the triplicates with different assays were pairwise plotted. The solid lines are trend lines and the dashed are the equality (*y* = *x*) lines.

The between-assay correlations of measurement results for the patient samples were further analyzed with pair-wise regressions. Because of the heteroscedasticity and the wide range of the data, Passing-Bablok regression was used for the estimation of slopes and intercepts of the assay pairs. The Pearson correlation coefficients were also calculated to get approximate indications of the linearity. The Passing-Bablok slopes and intercepts and the Pearson correlation coefficients are shown in **Table 3**. The slopes varied from 0.676 to 1.624 among the assay pairs. Though all the intercepts analytically seemed to be negligible, 3 of the 6 assay pairs showed intercepts that might be significantly different from 0. The Pearson correlation coefficients ranged from 0.956 to 0.989. The scatter plots for the assay pairs illustrating the linearity and the distribution of the data are presented in **Figure 1**.

**Table 3:** Between-assay correlations of measurement results for patient samples.

Assay pair (*x*–*y*)	Slope (95% CI)^a^	Intercept (95% CI)^a^	*r* b
Access-Architect	1.183 (1.099∼1.264)	−0.005 (−0.018∼0.008)	0.967
Access-Centaur	1.624 (1.523∼1.684)	−0.020 (−0.045∼−0.001)	0.989
Access-Vitros	1.068 (1.042∼1.122)	0.017 (0.003∼0.030)	0.974
Architect-Centaur	1.379 (1.223∼1.447)	−0.023 (−0.047∼0.006)	0.956
Architect-Vitros	0.957 (0.898∼0.994)	0.015 (−0.001∼0.026)	0.988
Centaur-Vitros	0.676 (0.631∼0.732)	0.031 (0.013∼0.052)	0.961

a Passing-Bablok slopes and intercepts expressed as mean and 95% confidence interval (CI).

b Pearson correlation coefficient.

### Commutability of Prepared Materials

**Figure 2:**
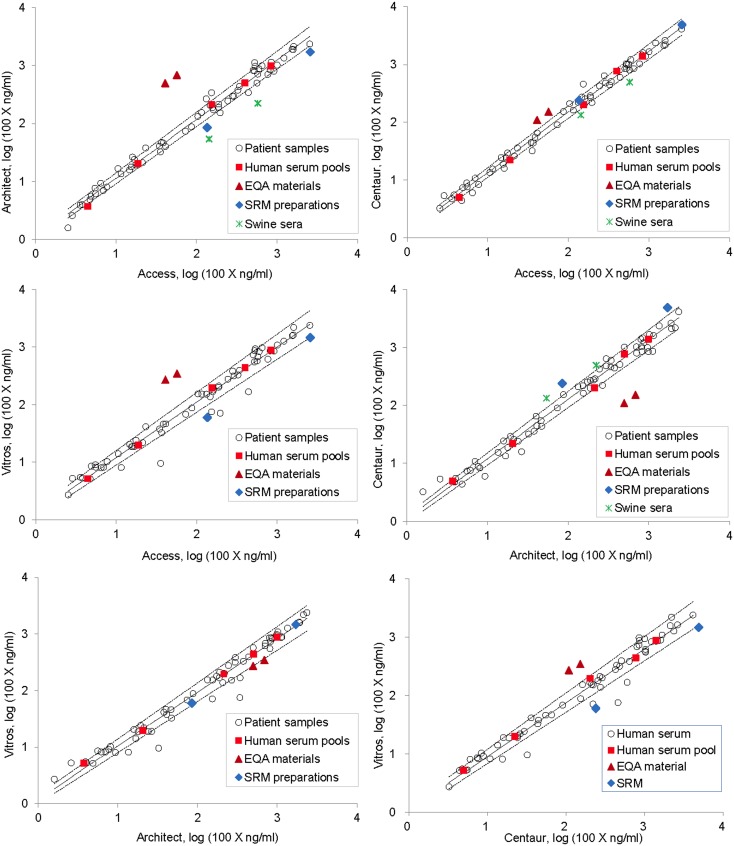
Commutability of prepared materials for cardiac troponin I (cTnI) measurement. Prepared materials (control materials used in our 2013 EQA program, frozen serum pools prepared from leftover patient samples, SRM preparations made by diluting the SRM 2921 with human serum and swine sera from MI model animals) together with a set of patient samples were measured for cTnI with 4 assays including Abbott Architect (Architect), Beckman Access (Access), Ortho Vitros (Vitros) and Siemens Advia Centaur (Centaur). The measurement results were logarithm-transformed and results for patient samples obtained with different assays were pairwise analyzed with Deming regression and 95% prediction intervals were calculated for each pair of assays. Prepared materials with measurement results (transformed) outside the prediction intervals are considered noncommutable. The solid lines are the regression lines and dashed are the limits of the prediction intervals.

The cTnI concentrations of the prepared materials (human serum pools, EQA materials, SRM preparations and swine sera) measured with different assays are listed in **Table 4**. Also because of the heteroscedasticity and the wide range of the data, logarithm transformation and Deming regression were used for the commutability evaluation. Commutability of the materials among different assays is shown in **Figure 2** and summarized in **Table 5**. The human serum pools were all commutable for all the assays. The SRM preparations were commutable for 2 of the 6 assay pairs. The EQA materials and the swine sera were all noncommutable for all the assays and the swine sera showed no reactivity to Vitros assay. To estimate the magnitude of the noncommutability of the materials, matrix-related biases were calculated (see the Materials and Methods section) for the noncommutable materials (**Table 6**). The matrix-related biases for the EQA materials ranged from −83% to 944%, the SRM preparations from −65% to 124%, and the swine sera from −68% to 99% (with the exclusion of Vitros assay).

**Table 4:** Measurement results for patient serum pools, EQA materials, SRM materials and swine sera.

	cTnI concentration, ng/ml										
	Human serum pools					EQA materials		SRM preparations		Swine sera	
Assay	L1	L2	L3	L4	L5	L2	L4	L1	L2	L1	L2
Access	8.41	4.05	1.56	0.19	0.04	0.57	0.41	25.80	1.36	5.79	1.43
Architec	9.89	5.02	2.14	0.21	0.04	6.87	4.97	17.11	0.85	2.23	0.54
Centaur	13.98	7.70	2.03	0.22	0.05	1.52	1.09	48.91	2.39	4.94	1.34
Vitros	8.86	4.41	1.97	0.20	0.05	3.45	2.71	14.72	0.60	ND^a^	ND^a^

a Not detectable.

**Table 5:** Commutability of patient serum pools, EQA materials, SRM preparations and swine sera.

Assay pair (*x*–*y*)	Patient serum pools	EQA materials	SRM preparations	Swine sera
Access-Architect	1	0	0	0
Access-Centaur	1	0	1	0
Access-Vitros	1	0	0	0
Architect-Centaur	1	0	0	0
Architect-Vitros	1	0	1	0
Centaur-Vitros	1	0	0	0

“1” and “0” denote commutable and noncommutable, respectively.

**Table 6:** Matrix-related biases for noncommutable materials.

	Commutability-related bias, %					
	EQA materials		SRM preparations		Swine sera	
Assay pair (*x*–*y*)	L2	L4	L1	L2	L1	L2
Access-Architect	931	944	−47	−47	−68	−68
Access-Centaur	83	85			−48	−39
Access-Vitros	439	483	−42	−59	NR^a^	NR^a^
Architect-Centaur	−83	−83	109	124	71	99
Architect-Vitros	−41	−37			NR^a^	NR^a^
Centaur-Vitros	209	231	−46	−65	NR^a^	NR^a^

a No reactivity of swine sera to Vitros assay.

**Figure 3:**
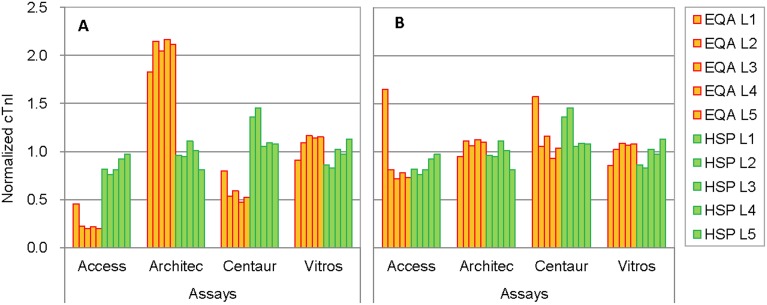
Between-assay variations on EQA materials before (A) and after (B) correction for matrix-related biases. The cTnI concentrations of 5 levels of EQA materials (EQA L1∼5) for different assays from a EQA program were normalized by dividing each assay group mean by the all-assay mean and the normalized values for EQA materials were compared with that for human serum pools (also 5 levels, HSP L1∼5). Between-assay variations on the EQA materials were much larger (CV of ∼80%) than that on the human serum pools (CV of ∼20%) because of matrix-related biases (A). The variations became similar when the biases were corrected (B).

The matrix-related biases of the EQA materials definitely caused abnormal between-assay variations in the EQA program. **Figure 3A** shows normalized cTnI levels (values relative to the all-assay mean) of the EQA materials (level 1∼5, calculated from Table 1 data) in comparison with that of the human serum pools (from Table 4). Between-assay variations on the EQA materials were much larger (CV of ∼80%) than that on the human serum pools (CV of ∼20%). Correction for the matrix-related biases was tried. The normalized cTnI values of EQA material level 2 and human serum pool level 3, which were the respective medians of the 5 levels of the 2 categories of samples, were compared and correction factors were calculated for each assay by dividing the human serum pool value by the EQA material value. The factors were applied to all EQA values and the between-assay variations became similar to that on human serum pools as shown in **Figure 3B**.

## Discussion

Assays for cTnI measurement have evolved several generations and improved considerably in respects of analytical sensitivity, precision and between-assay variation [2], [3]. The improvement will continue as more efforts are being made toward standardization of cTnI measurement [2], [4], [5]. The performance of cTnI measurement is most conveniently monitored through EQA programs. For EQA programs to fulfill this purpose, however, EQA materials need to be commutable or to be of known commutability [8].

There have been very few studies on the commutability of materials for cTnI measurement [5], [13]. Candás-Estébanez et al [19] reported apparently different measurement precisions on control materials and plasma samples measured with a cTnI measurement system. Information on the commutability of possible EQA materials is basically lacking. In this study, we evaluated the commutability of commercial control materials, human serum pools prepared from patient samples, purified analyte preparations and model animal serum samples, which would represent major possible sources of EQA materials. The study showed that only human serum pools were commutable among major cTnI assays and all other materials were variously noncommutable with matrix-related biases ranging from −83% to 944% (**Figure 2**, **Table 5** and **Table 6**).

The commutability of human serum pools has been suggested by their ability to harmonize measurement results of different assays [5], [12]. The commutability may also be assumed because the pools can be considered averaged patient samples. However, preparation of the pools requires multiple treatments of patient samples and it is important to test whether the treatments, especially the freeze-thawing, diluting with normal serum, filtering and prolonged storage at various temperatures, causes alterations in the analyte or its matrix that influence the measurement. This study demonstrates that cTnI is resistant to the treatments and commutable sample materials can be prepared from leftover patient samples.

The measurement of cTnI seems to be especially susceptible to commutability influences. In this study, swine sera showed no reactivity to one assay, and matrix-related biases as high as ∼10 fold were observed on the control materials, and even the SRM preparations, the analyte of which is human troponin complex, showed matrix-related biases of up to ∼2 fold (**Table 6**). The causes of the noncommutability are complicated and related to both the measurement principles of the assays and nature of the analyte and its matrix [3]. Current cTnI assays are sandwich type immunoassays using monoclonal capture and detection antibodies. Different assays may use different combinations of antibodies with various specificities and affinities to the cTnI molecule. It is known that serum cTnI is subject to posttranslational modifications, such as proteolytic degradation and phosphorylation, and complexations with other molecules (e.g., TnC, heparin, heterophile antibodies, and cTnI specific autoantibodies) in the circulation [3]. For this and probably other reasons, the cTnI analytes in the prepared materials may be different from patient serum cTnI, depending on the origin and the history of processing of the materials. Animal cTnI may also be different in primary structure [15], [16]. Furthermore, the matrixes of the control materials and the swine sera would apparently be different from that of patient serum. All these differences may influence different assays to varying degrees and the observed noncommutability would be a reflection of the variable influences.

The complexity of cTnI measurement would also be reflected by the between-assay correlations of measurement results of patient samples (**Figure 1** and **Table 3**). Relatively large scatters and low Pearson correlation coefficients of the data were observed on the assay pairs.

The noncommutability of the control materials caused exceptional between-assay variations in our EQA programs (**Table 1** and **Figure 3A**). The matrix-related biases on the materials ranged from −83% to 944% (**Table 6**). Without this information, the EQA results could hardly be interpreted and might lead to erroneous conclusions regarding the comparability of the assays.

EQA programs should ideally use commutable sample materials. Based on the results of this study and other available information [2]–[5], [12], [13], human serum pools prepared from patient samples seem currently to be the only commutable materials for cTnI measurement. EQA programs desirably distribute multi-level samples that cover a large part of the measurement ranges of assays. Obviously, it is difficult to prepare all the EQA materials from patient samples. Theoretically, EQA programs can also use sample materials of known commutability. However, commutability is highly material and assay specific and it is almost impossible to test the materials with all contemporary assays for all EQA events. A practical approach may be a combination of human serum pools with other more easily available materials. An EQA program includes at least one human serum pool that has an analytically relevant cTnI value, and uses other materials for all other intended sample levels. The EQA process itself serves as a “commutability study” at the same time and the matrix-related biases can be reasonably corrected by applying factors to the assay peer group means as described in the Results section and shown in **Figure 3B**. Similar approaches have also been proposed in previous reports [8]. This correction is obviously based on an assumption that the matrix-related biases are proportional and thus the materials should be of the same origin. The noncommutability of the materials would also need to be reasonable, or the inter-laboratory CV for the peer groups may not be reliable. For this purpose, human troponin TIC complex diluted with human serum would be possible candidates based on this study. The usefulness of swine serum materials is uncertain and depends on monoclonal antibodies used in future assays.

It should also be noted that the ideal EQA is that in which, besides the use of commutable samples, the values of the samples are assigned with a reference method or an accepted protocol so that all the routine assays can be evaluated against “true values” [8]. This has actually been realized for some clinical chemistry analytes, such as cholesterol, creatinine, and HbA1c. International organizations are working on the reference measurement of cTnI [20], [21] and accuracy-based EQA for cTnI may be expected in the future.

A major limitation of the study is that only 4 assays were used for the commutability evaluations. This is mainly because of the available volume of individual patient samples. It is very difficult to get sufficient volume of individual samples for measurement in triplicate with more assays. Another limitation is that the patient samples were frozen before analysis. This is related to the availability of sufficient number of fresh cTnI positive samples with sufficient leftover volumes. It took about 2 months to collect the 75 samples in 2 hospitals. The commutability of human serum pools demonstrated in the study may imply the acceptability of the use of frozen samples.

In conclusion, commutability of EQA materials has major impact on the effectiveness of EQA programs for cTnI measurement. Human serum pools prepared from patient samples are commutable and other materials are mostly noncommutable. EQA programs for cTnI should include at least one human serum pool to allow proper interpretation of EQA results.

## Supporting Information

Table S1 Raw data of the study and major data analyses.
(XLSX)
